# A Familial X‐Linked Disorder of Sexual Development in Thoroughbred Horses Associated With a Novel Androgen Receptor Splice‐Site Variant

**DOI:** 10.1002/age.70112

**Published:** 2026-04-26

**Authors:** Anna Letko, Vidhya Jagannathan, Cord Drögemüller, Gesine Lühken

**Affiliations:** ^1^ Institute of Genetics, Vetsuisse Faculty University of Bern Bern Switzerland; ^2^ Institute of Animal Breeding and Genetics Justus Liebig University Giessen Giessen Germany

Differences or disorders of sexual development (DSD) have been described in various animal species for decades (OMIA:000564). DSD encompass a range of congenital conditions characterized by various clinical signs, such as infertility and impaired development of sexual characteristics. The variability is reflected in the heterogeneity of molecular causes and modes of inheritance described for different forms of DSD (Basrur 2006). Androgen insensitivity syndrome (AIS) is an X‐linked recessive DSD in which affected males present with undescended testes and female secondary sexual characteristics (XY DSD; OMIM:300068; and OMIA:000991) (Delli Paoli et al. 2023). In horses, five likely causal variants for AIS in the equine androgen receptor (*AR*) gene have been previously described in different breeds (Villagomez et al. 2020) (Figure 1a), including two detected in the Thoroughbred breed (OMIA Variant IDs 786 and 1144). In addition, a large deletion on equine chromosome 29 has been characterized as a risk factor for reproductive disorders, including DSD (Ghosh et al. 2020).

**FIGURE 1:**
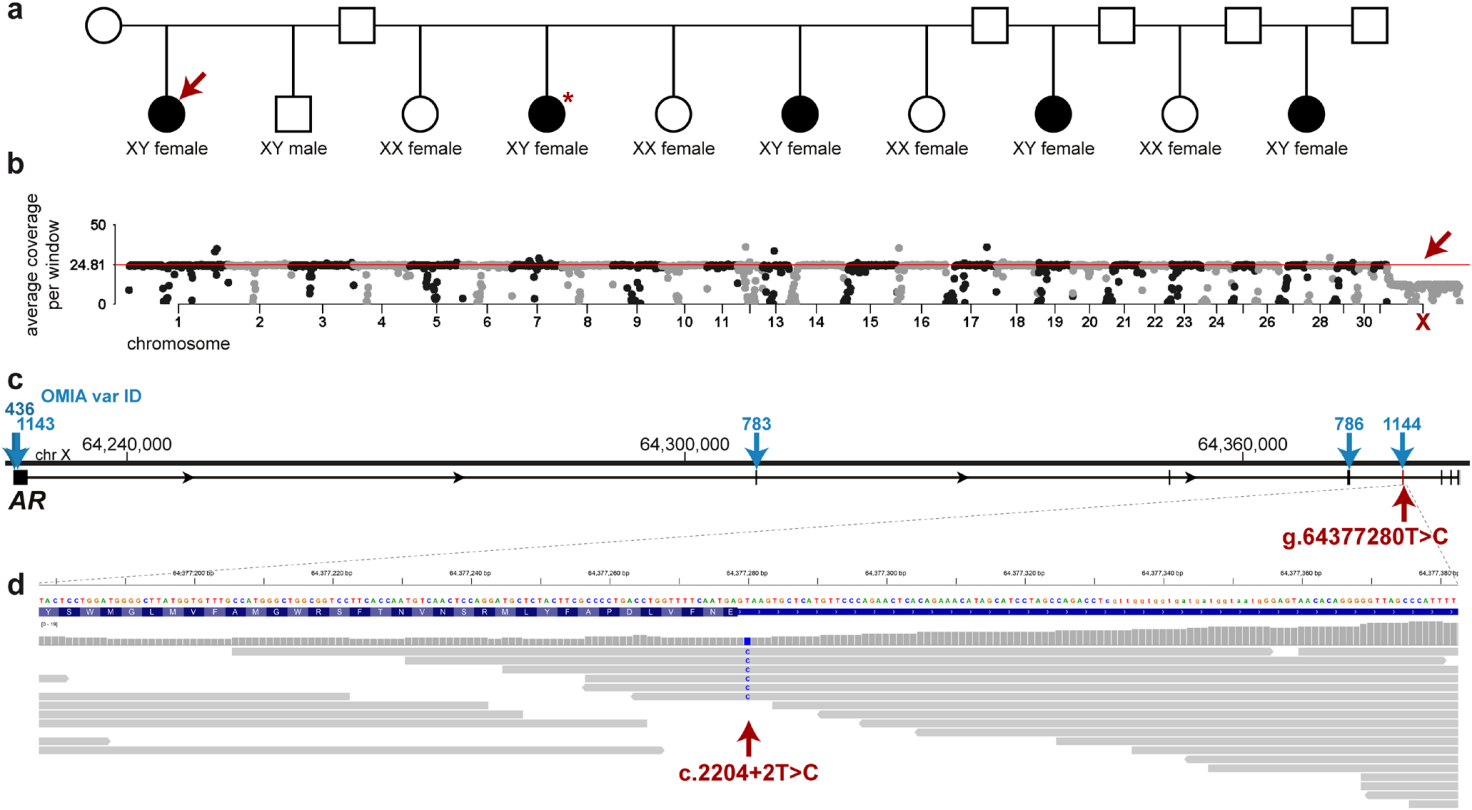
Identification of an *AR* variant on chromosome X in a Thoroughbred horse showing a form of disorder of sexual development. (a) Pedigree of the studied Thoroughbred family, red arrow shows the whole‐genome sequenced case, and red asterisk indicates the 64, XY karyotyped case; phenotypically males are shown as squares, females as circles. The five phenotypically female and infertile horses, suspected to be genetically male (XY), are indicated with black‐filled circles. (b) Plot of read depth averaged over 1‐Mb windows across the genome of the affected female, average genome coverage of 24.81 is shown as red line. Note the decrease of coverage of the entire X chromosome (red arrow), indicating only one copy and 64, XY karyotype. (c) Schematic representation of the *AR* gene with locations of five previously described equine variants reported in OMIA (blue arrows, numbers correspond to the OMIA var. IDs) and the newly identified candidate variant (red arrow). (d) IGV screenshot of the affected horse showing the candidate variant affecting splice donor site of exon 5 of the *AR* gene.

This study looked at a Thoroughbred racehorse family with the presence of XY DSD animals. Of the 10 foals born over a period of 15 years from one mare that was mated with five different stallions, only one was phenotypically male (Figure 1a). Previously, one of this mare's offspring, which was phenotypically female, was identified as genetically male (XY) by karyotyping (indicated by asterisk in Figure 1a) after testing positive for testosterone in a race, which raised suspicions of DSD. Three other female offspring were reported infertile and suspected XY DSD, but were not investigated further, whereas four additional females proved to be fertile (Figure 1a). For this study, genomic DNA of the mare and the most recently reported phenotypically female foal (indicated by the arrow in Figure 1a) was isolated from EDTA‐blood samples. Collection of the samples was approved by the Veterinary Department of the Regional Council of Giessen (19 c 20 15 h 02 Gi 19/1 KTV 22/2020). The case was tested positive by PCR for the presence of fragments from the Y‐chromosomal genes *SRY* and *NLGN4Y* (Zaffalon et al. 2019). The presence of either of the two already known AR variants for AIS in Thoroughbred horses (OMIA Variant IDs 786 and 1144) was excluded in the case by Sanger sequencing of PCR fragments amplified with primer pairs published previously (Villagomez et al. 2020; Bolzon et al. 2016).

Subsequently, short‐read whole‐genome sequencing (WGS) was performed in the case on Illumina NovaSeq6000. The obtained reads were aligned to the TB‐T2T reference assembly (GCF_041296265.1) as previously described (Jagannathan et al. 2019) and resulted in 24.81× average genome coverage. Variant calling was performed using the Genome Analysis Toolkit best practices (Van der Auwera and O'Connor 2020) and 107 publicly available horse genomes of various breeds were used as controls (Table S1) for filtering of variants private to the case. Functional effects of the called variants were predicted with SnpEff v5.0c (Cingolani 2022) and high or moderate impact variants were considered protein‐changing. Further prioritization based on gene function was performed with the VarElect tool (Stelzer et al. 2016). Integrative Genomics Viewer (IGV) (Thorvaldsdóttir et al. 2013) was used for visual inspection of the detected variants. None of the previously described AIS‐related variants were found in the case. Genome‐wide read depth averaged over 1 Mb windows was plotted and did not show any evidence for larger structural variants in the autosomal genome (Figure 1). The phenotypically female horse showed reduced coverage of the entire X chromosome confirming the 64, XY karyotype (Figure 1b). Out of the total 5 791 894 variants called in the case, 65 378 were present only in the affected horse and absent from the 107 controls. Further filtering identified 790 private protein‐changing variants: 2 hemizygous, 13 homozygous, and 775 heterozygous (Table S2). Only one of them was located in a functional candidate gene on chromosome X, the *AR* gene. This splicing variant (NC_091715.1:64377280T>C) was predicted to affect the splice donor site of exon 5 (NM_001163891.1:c.2204+2T>C; Figure 1c,d) by the SpliceAI tool (Jaganathan et al. 2019) with a probability of 98%. The phenotypically normal mare was confirmed as heterozygous carrier by Sanger sequencing of a PCR product amplified with the primer pair 5′‐TCTCCCCCTGCTCTCCTAAC‐3′ and 5′‐GCTTTAGCACCAACCCTCCT‐3′. Additionally, to investigate possible segregation within the global Thoroughbred population at low frequency, three publicly available variant catalogues (including 340 Thoroughbred and 480 horses of other breeds) (Tozaki et al. 2021; Durward‐Akhurst et al. 2021; Bailey et al. 2024) were queried for the candidate variant, which was absent from all of them.

The affected *AR* gene encodes a protein that functions as a steroid‐hormone‐activated transcription factor that is critical for the development and maintenance of male sexual characteristics (Nitsche and Hiort 2000). Several *AR* knockout mice models for AIS are described with reproductive system abnormalities (MGI:88064). In humans, more than 300 AIS‐associated pathogenic and likely pathogenic variants in *AR*, including 12 splice‐site variants, are currently reported in the ClinVar database (2025). The herein described equine *AR* variant likely results in a loss of the canonical splice donor site. Unfortunately, RNA samples from the affected horse were not available for further investigation. However, given the functional importance of the gene and similarity of the disease phenotype across species, the candidate variant is considered causal for the observed cases of X‐linked recessive AIS in the Thoroughbred. Given the absence of the alternative allele in 927 control genomes, the variant likely arose *de novo* in the dam. However, this could not be confirmed experimentally due to the unavailability of parental samples. In Germany, the observation of XY DSD in the active racing horse from the herein described family led to relaxation of existing rules and the permitting of DSD horses to participate in races, albeit only in those designated for stallions.

## Author Contributions

**Anna Letko:** conceptualization, formal analysis, investigation, visualization, writing – original draft, writing – review and editing. **Vidhya Jagannathan:** formal analysis, data curation. **Cord Drögemüller:** methodology, resources, writing – review and editing. **Gesine Lühken:** project administration; resources; writing – review and editing.

## Funding

The authors have nothing to report.

## Conflicts of Interest

The authors declare no conflicts of interest.

## Supporting information


**Table S1:** List of all 108 horse genomes of various breeds sequenced as part of the ongoing Swiss Comparative Equine Resequencing project.

**Table S2:** List of private variants in the affected horse obtained after the comparison to 107 horse genomes of different breeds.

## Data Availability

The data that support the findings of this study are openly available in European Nucleotide Archive at https://www.ebi.ac.uk/ena/browser/home, reference number SAMEA120226917.
